# Effects of Stem Cell Factor/c-Kit Signaling on *In Vitro* Maturation of Porcine Oocytes and Subsequent Developmental Competence After Fertilization

**DOI:** 10.3389/fvets.2021.745488

**Published:** 2021-10-08

**Authors:** Eunhye Kim, Lian Cai, Sang-Hwan Hyun

**Affiliations:** ^1^Laboratory of Veterinary Embryology and Biotechnology, Veterinary Medical Center and College of Veterinary Medicine, Chungbuk National University, Cheongju, South Korea; ^2^Graduate School of Veterinary Biosecurity and Protection, Chungbuk National University, Cheongju, South Korea

**Keywords:** oocyte maturation, porcine oocytes, pre-implantation embryonic development, SCF/kit signaling, stem cell factor

## Abstract

Stem cell factor (SCF), also known as c-Kit ligand, plays an important role in the proliferation of primordial germ cells and the survival of oocytes during follicular development. The aim of this study was to investigate the effect of SCF/c-Kit signaling on *in vitro* maturation (IVM) of porcine oocytes by analyzing nuclear and cytoplasmic maturation, oocyte size, cumulus cell expansion, and developmental competence to the blastocyst stage. Moreover, mRNA expression patterns of porcine cumulus cells and oocytes were evaluated using qRT-PCR. Following 42 h of IVM, 10 and 50 ng/mL SCF-treated groups exhibited significantly (*P* < 0.05) increased polar body extrusion rates and intracellular glutathione levels compared with the control group. The cumulus expansion index significantly (*P* < 0.05) increased in all SCF-treated groups compared with the control samples. mRNA levels of the proapoptotic gene *Bax* and apoptosis-related cysteine peptidase *Caspase3* were lower in SCF-treated cumulus cells than in the control group. Notably, the diameter of oocytes after IVM, the mRNA expression of well-known oocyte-secreted factors (*GDF9* and *BMP15*), and an oocyte-specific protein essential for ovulation and oocyte health (*YBX2*) were significantly (*P* < 0.05) higher in SCF-treated than in non-treated oocytes. Inhibition of c-Kit during porcine IVM using ACK2, an antagonistic blocker of c-Kit, significantly (*P* < 0.05) decreased the polar body extrusion rate compared with the control, as well as blastocyst formation rate compared with the 10 ng/mL SCF-treated group. In conclusion, the effect of SCF/c-Kit-mediated signaling during porcine IVM could be ascribed to the reduced expression of apoptosis-related genes and higher expression of oocyte-specific/secreted factors.

## Introduction

In mammals, bi-directional cellular communication between oocytes and neighboring granulosa cells is essential for follicular development (1). Various cytokines and growth factors are released from each follicular compartment, which affects the functioning of each other (2). One of the important ligand-receptor systems that mediate granulosa-oocyte interactions is the c-Kit, a tyrosine kinase receptor, and its ligand (KITL), the stem cell factor (SCF) (3). SCF is a cytokine that binds to c-Kit, also known as CD117, which plays an important role in early hematopoiesis (4). At most stages of follicular development, c-Kit continues to be expressed on oocyte surfaces, whereas SCF is expressed by granulosa cells in various mammalian species, including mice (5), humans (6), and pigs (7)). The SCF/c-Kit signaling pathway stimulates the initiation of primordial follicle development (8), enhances growth and survival of oocytes (9, 10), reawakens dormant oocytes (11), and plays a role in antrum formation and steroidogenesis *in vivo* (12).

Mammalian oocytes matured *in vitro* are known to have reduced developmental competence compared with matured oocytes *in vivo*, owing to the loss of follicular environment. SCF production *in vitro* is far reduced as compared with that *in vivo* as SCF expression is highly dependent on the activity of the follicle stimulating hormone (13, 14), suggesting that SCF supplementation is necessary *in vitro*. Therefore, some investigators have attempted to improve *in vitro* maturation (IVM) systems by adding SCF to cultures of murine (15), bovine (16), and buffalo (17) oocytes. However, data cannot be directly extrapolated into porcine oocytes because several differences exist between various mammalian species and their effects on oocyte maturation remain controversial; for example, porcine IVM takes >40 h, unlike IVM of other mammal oocyte cultures. In bovine oocytes, addition of SCF during IVM had no effect on nuclear maturation; however, it had a positive effect on cytoplasmic maturation and developmental competence (16). In contrast, in murine oocytes, addition of SCF during IVM promoted the first polar body extrusion; however, it had no impact on cytoplasmic maturation (15). In buffalo, SCF treatment during IVM increased nuclear maturation and viability of oocytes (17). Nonetheless, its role in *in vitro* growth and maturation of porcine oocytes remains unclear.

Given that SCF is expressed by granulosa cells and that it is positively correlated with ovarian steroidogenesis in pigs (18), we hypothesized that supplementation of SCF during IVM could have a positive effect on the development of *in vitro* porcine oocytes that are separated from the follicular environment. In the present study, we examined the effect of SCF on nuclear maturation, intracellular levels of glutathione (GSH), and reactive oxygen species (ROS) in porcine oocytes, and cumulus expansion. Additionally, we investigated the effect of inhibition of SCF/c-Kit signaling during porcine IVM using ACK2, a monoclonal c-Kit neutralizing antibody, that acts as an antagonist of c-Kit function (19).

## Materials and Methods

### Chemicals

All chemicals and reagents used in this study were purchased from Sigma-Aldrich (St. Louis, MO, USA) unless otherwise mentioned.

### Oocyte Collection and *in vitro* Maturation

Ovaries were collected at a local slaughterhouse and transported to the laboratory within 2 h in 0.9% (w/v) NaCl solution containing 100 IU/L penicillin G and 100 mg/mL streptomycin sulfate at 35°C. *In vitro* oocyte collection and maturation was performed as previously described (20). Cumulus oocyte complexes (COCs) in ovaries were aspirated from 3 to 6 mm diameter superficial follicles using an 18 gauge needle attached to a 10 mL disposable syringe. Following settling at 37°C for 5 min, the precipitate was resuspended in HEPES-buffered Tyrode's medium containing 0.05% (wt/vol) polyvinyl alcohol (TLH-PVA) and examined using a stereomicroscope to recover the COCs. Only COCs with ≥3 uniform layers of compact cumulus cells and uniform ooplasm were selected. Approximately 60 COCs were placed into each well of a 4-well-dish (Nunc, Roskilde, Denmark) containing 500 μL of culture medium (TCM199; Invitrogen, Waltham, MA, USA) supplemented with 0.6 mM cysteine, 0.91 mM sodium pyruvate, 10 ng/mL epidermal growth factor, 75 μg/mL kanamycin, 1 μg/mL insulin, 10 IU/mL equine chronic gonadotropin (eCG), 10 IU/mL human chorionic gonadotropin (Intervet, Boxmeer, Netherlands), and 0.1% (w/v) PVA. For IVM, selected COCs were incubated at 39°C with 5% CO_2_ and 95% air in a humidified chamber. COCs were incubated for ~42 h, of which, the first 22 h were in media with the eCG hormone, and the following 20 h of incubation were without the hormone. COCs were treated with or without SCF, and SCF was added to the medium throughout the entire maturation period.

### Evaluation of Nuclear Maturation, Cumulus Cell Expansion Index, and Oocyte Diameter

Following maturation, cumulus cells were removed by gentle pipetting with 0.1% hyaluronidase in IVM medium and washed in TLH-PVA. Oocytes with first polar body extrusion were regarded as mature oocytes. The experiment was performed in triplicate. To evaluate the extent of cumulus cell expansion, post-IVM COCs from each group were visualized under an optical microscope, and images were acquired. The cumulus cell expansion index (CEI) after 22 h of IVM was calculated using a previously described method (21). The cumulus cell expansion of porcine COCs after 42 h of IVM was measured using three steps based on previously described scales (22, 23). Diameter of porcine oocytes was calculated according to the method described in a previous report (24), as follows:


$$\begin{array}{l} \text{Porcine oocyte diameter}=\left({\text{OOC}}_{1}+{\text{OOC}}_{2}\right)/2. \end{array}$$

### Measurement of Intracellular GSH and ROS Levels

To determine intracellular GSH and ROS levels, oocytes were sampled 42–44 h after IVM. GSH and ROS levels were assessed as previously described (25, 26). Briefly, 2′,7′-dichlorodihydrofluorescein diacetate (H2DCFDA; Invitrogen) and 4-chloromethyl-6.8-difluoro-7-hydroxycoumarin (CellTracker Blue; CMF2HC; Invitrogen) were used to detect intracellular ROS (green fluorescence) and GSH (blue fluorescence), respectively. Ten oocytes from each group were incubated in the dark for 30 min in TLH-PVA supplemented with 10 μM H2DCFDA and 10 μM CellTracker Blue. Afterwards, the oocytes were washed with fresh TLH-PVA and transferred to a 10 μL drop, which was used for fluorescence measurement with an epifluorescence microscope (TE300; Nikon, Tokyo, Japan) with UV-2A (370 nm for GSH) and GFP-B (460 nm for ROS) filter. The fluorescence intensity of the oocytes from each treatment group was analyzed using ImageJ software (Version 1.41; National Institutes of Health, Bethesda, MD, USA) and normalized to that of the control group. The experiment was performed in triplicate (GSH samples, *n* = 240; ROS samples, *n* = 197).

### *In vitro* Fertilization and Culture

*In vitro* fertilization (IVF) was performed as described previously (20). Briefly, liquid semen supplied weekly from the Veterinary Service Laboratory (Department of Livestock Research, Yong-in City, Gyeonggi-do, Republic of Korea) was stored at 17°C for 5 days before use. The sample was washed twice with Dulbecco's phosphate-buffered saline (DPBS) supplemented with 0.1% bovine serum albumin *via* centrifugation at 2,000 × g for 2 min. Following washing, the sperm pellet was resuspended in modified Tris-buffered medium (mTBM) (27), which was pre-equilibrated for 18 h at 39°C in 5% CO_2_ and 95% air. Following appropriate dilution, a 5 μL sperm suspension was added to a 40 μL drop of mTBM containing 15 mature porcine oocytes for a final sperm concentration of 1 × 10^6^ sperm/mL. Sperm motility was assessed prior to fertilization, and >80 % motile sperm were used in each experiment. Oocytes were coincubated with sperm for 20 min at 39°C in a humidified atmosphere of 5% CO_2_ and 95% air. Thereafter, loosely attached sperm cells were removed from the zona pellucida *via* gentle pipetting. Following washing, oocytes were incubated in mTBM without sperm for 5–6 h at 39°C in a humidified atmosphere of 5% CO_2_ and 95% air. Thereafter, presumptive zygotes were washed and cultured in 25 μL microdrops (10 gametes/drop) of porcine zygote medium 3 (PZM3) (28) at 39°C for 168 h under a humidified atmosphere of 5% O_2_, 5% CO_2_, and 90% N_2_. The medium was changed every 2 days. To evaluate the developmental competence, Day 0 was regarded as the day on which IVF was initiated. Day 2 after IVF, the cleavage rates were analyzed and embryos were categorized into three groups; 2–3, 4–6, and 7–8 cells. Day 7 after IVF, blastocyst formation rates were measured and the blastocysts were categorized into three groups according to degree of expansion and hatching status: early, expanded, and hatched, as previously described (20). The experiments were repeated three times.

### Gene Expression Analysis *via* Quantitative Real-Time Polymerase Chain Reaction

To analyze gene expression in SCF-treated mature oocytes and cumulus cells after IVM, oocytes, and cumulus cells were separately isolated from 60 COCs each using 0.1% hyaluronidase and sampled using a stereomicroscope. All samples were washed twice in DPBS and stored at −80°C until analysis. Expression of *Has2, Bax*, Caspase 3 (*Cas3*), growth differentiation factor-9 (*GDF9*), and Y-box-binding protein-2 (*YBX2*) was measured in cumulus cells, and that of *c-kit*, AKT serine/threonine kinase-1 (*Akt1*), mammalian target of rapamycin (*mTOR*), *GDF9*, bone morphogenetic protein-15 (*BMP15*), and *YBX2* was measured in oocytes. Primer sequences used for qRT-PCR are presented in Table 1. Total RNA was extracted using TRIzol reagent (TaKaRa Bio, Kusatsu, Japan) according to the manufacturer's protocol. Complementary DNA (cDNA) was synthesized from the extracted mRNA using Moloney murine leukemia virus reverse transcriptase (Elpis-Biotech, Daejeon, Korea) and random primers (6-mer; Takara Bio), 10 mM dNTPs (BEAMS Bio Technologies, Seongnam, Korea), and RNase inhibitor (Intron Biotechnology, Seongnam, Korea). Synthesized cDNA was mixed with 2 × SYBR Premix Ex Taq (Takara Bio) to perform qRT-PCR (Mx3000P qRT-PCR, Agilent Technologies, Santa Clara, CA, USA) with 10 pmol of specific primers. Reactions were performed in 40 cycles, and cycling parameters were as follows: denaturation at 95°C for 15 s, annealing at 57°C for 15 s, and extension at 72°C for 15 s. Fluorescence intensity was measured at the end of the extension phase of each cycle. The threshold value for fluorescence intensity of all samples was set manually. The reaction cycle at which the PCR products exceeded this fluorescence intensity threshold was defined as the threshold cycle (Ct) in the exponential phase of PCR amplification. The relative expression (R) was calculated using the following equation: R = 2^−[Δ*Ctsample*−Δ*Ctcontrol*]^. To determine a normalized R-value for each gene, every obtained value was normalized to that of *GAPDH* and *RN18S* in cumulus cells and oocytes, respectively. The experiments were performed in triplicate.

**Table 1:** Primer sequences for qRT-PCR.

**mRNA**	**Primer sequences**	**Product size (bp)**	**GenBank accession number**
*RN18S*	F: 5′-CGCGGTTCTATTTTGTTGGT-3′	219	NR_046261.1
	R: 5′-AGTCGGCATCGTTTATGGTC-3′		
*GAPDH*	F: 5′-GTCGGTTGTGGATCTGACCT-3′	207	NM_001206359.1
	R: 5′-TTGACGAAGTGGTCGTTGAG-3′		
*c-kit*	F: 5′-GGGAGGATTATCCCAAGTCT-3′	127	NM_001044525.1
	R: 5′-GAATTGACATCAGCATTGGA-3′		
*AKT1*	F: 5′-CTACAACCAGGACCACGAGA-3′	208	NM_001159776.1
	R: 5′-CTCATACACATCCTGCCACA-3′		
*mTOR*	F: 5′-CACTCGCTCTTTAGCAGTCA-3′	214	XM_003127584.6
	R: 5′-ACCAGTGACCTCCATAGCAT-3′		
*GDF9*	F: 5′-GGTTCCAGCTTCATTCAATC-3′	120	NM_001001909.1
	R: 5′-ACAATCCAGTTGTCCCACTT-3′		
*BMP15*	F: 5′-CCATCATCCAGAACCTTGTC-3′	154	NM_001005155.2
	R: 5′-CAGGACTGGGCAATCATATC-3′		
*YBX2*	F: 5′-TCCTCCCCTTTCCCATAATC-3′	187	XM_021067811.1
	R: 3′-GTTCCTTCTCAGCCTGATCG-3′		
*Has2*	F: 5′-TTACAATCCTCCTGGGTGGT-3′	199	NM_214053.1
	R: 5′-TCAAGCACCATGTCGTACTG-3′		
*Bax*	F: 5′-TGCCTCAGGATGCATCTACC-3′	199	XM_003127290
	R: 5′-AAGTAGAAAAGCGCGACCAC-3′		
*Cas3*	F: 5′-CGTGCTTCTAAGCCATGGTG-3′	186	NM_214131
	R: 5′-GTCCCACTGTCCGTCTCAAT-3′		

### Experimental Design

In experiment 1, the effects of treatment with various concentrations (0, 5, 10, and 50 ng/mL) of SCF during IVM on porcine COCs were examined to identify the optimal concentration. For this, cumulus cell expansion index and mRNA expression of the cumulus expansion-related gene (*Has2*) and apoptosis-related genes (*Bax* and *Cas3*) were analyzed in cumulus cells. Moreover, polar body extrusion rates and size were measured in oocytes. Furthermore, intracellular levels of GSH and ROS were investigated.

In experiment 2, the effects of SCF/c-Kit signaling during IVM were examined. The role of c-Kit in the porcine IVM system has been demonstrated by inhibiting experiments using its specific antibody, ACK2. Hence, cumulus expansion, polar body extrusion rates, subsequent embryonic development of IVF embryos, and mRNA expression patterns in cumulus cells and oocytes were investigated. In oocytes, the mRNA expression of SCF/c-Kit signaling-related genes (*c-kit, Akt*, and *mTOR*) and that of oocyte secreted factors (*GDF9, BMP15*, and *YBX2*) were analyzed. In cumulus cells, the mRNA expressions of the cumulus expansion-related gene (*Has2*), apoptosis-related genes (*Bax* and *Cas3*), and oocyte secreted factors (*GDF9* and *YBX2*) were analyzed.

### Statistical Analysis

Statistical analyses were performed using Prism version 8.0 (GraphPad Software, San Diego, CA, USA). Results are expressed as mean ± SEM. Experiments were repeated at least three times, unless stated otherwise. Percentage data (that is, polar body extrusion rates, cleavage rate, and blastocyst formation rate) were analyzed using ANOVA. Statistical significance was set at *p* < 0.05. Statistical methods, *p*-values, and sample numbers are indicated in each figure legend.

## Results

### Effect of SCF Supplement During Porcine IVM on Cumulus Cell Expansion

**Figure 1:**
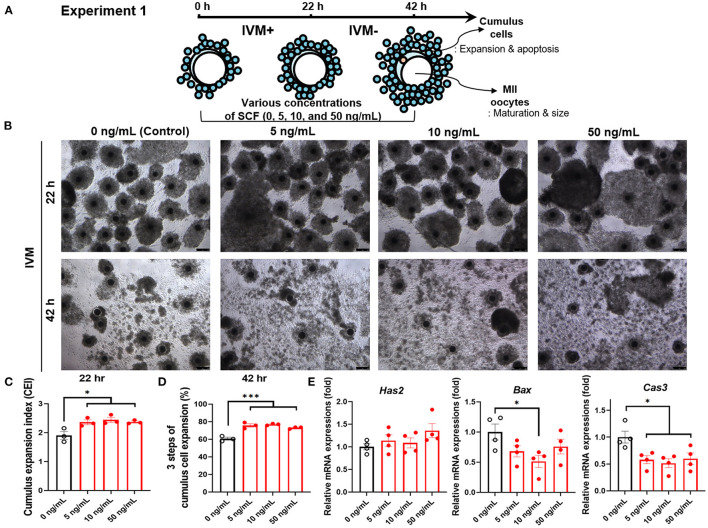
Effect of stem cell factor (SCF) supplementation during *in vitro* maturation (IVM) on cumulus cell expansion of porcine cumulus-oocyte complexes (COCs). **(A)** Experimental design to determine the optimal concentrations of SCF for porcine oocyte maturation. IVM+ and IVM– indicate that incubation was in media with or without the eCG hormone, respectively. **(B)** Representative morphologies of porcine COCs from each group after 22 or 42 h of IVM supplemented with various concentrations of SCF. Scale bar = 100 μm. **(C)** Quantified cumulus expansion index (CEI) after 22 h of IVM supplemented with various concentrations of SCF. **(D)** Quantified three steps of cumulus cell expansion after 42 h of IVM supplemented with various concentrations of SCF. **(E)** The mRNA expression levels of the genes *Has2*, related to cumulus cell expansion, *Bax*, and *Caspase 3* (*Cas3*), related to apoptosis, were compared between cumulus cells from various SCF-treated groups. For all graphs, the value represents the mean ± SEM. Asterisks indicate statistical significance (*p < 0.05 and ***p < 0.001). Statistical significance was determined by one-way ANOVA. The experiments were repeated four times.

For experiment 1, as shown in Figure 1A, porcine oocytes matured in IVM medium supplemented with various concentrations of SCF (5, 10, and 50 ng/mL) for 42 h. Thereafter, we evaluated the effects of treatment with different concentrations of SCF during porcine IVM on cumulus expansion. As shown in Figure 1B, compared with the mature COCs of the control group, those of all SCF-treated groups showed increased cumulus CEI at 22 h (Figure 1C) and percentage of three steps of cumulus cell expansion at 42 h (Figure 1D). Following 42 h of IVM, we investigated the expression of genes related to cumulus cell expansion and apoptosis in porcine cumulus cells. Although no significant changes (*p* > 0.05) in the mRNA levels of *Has2* (hyaluronan synthase 2) were observed, the 10 ng/mL SCF-treated group displayed significantly decreased mRNA levels of both apoptosis-related *Bax* and *Cas3* (*p* < 0.05) transcripts compared with the control (Figure 1E).

### Effect of SCF Supplement During Porcine IVM on Nuclear and Cytoplasmic Maturation and Size of Oocytes

**Figure 2:**
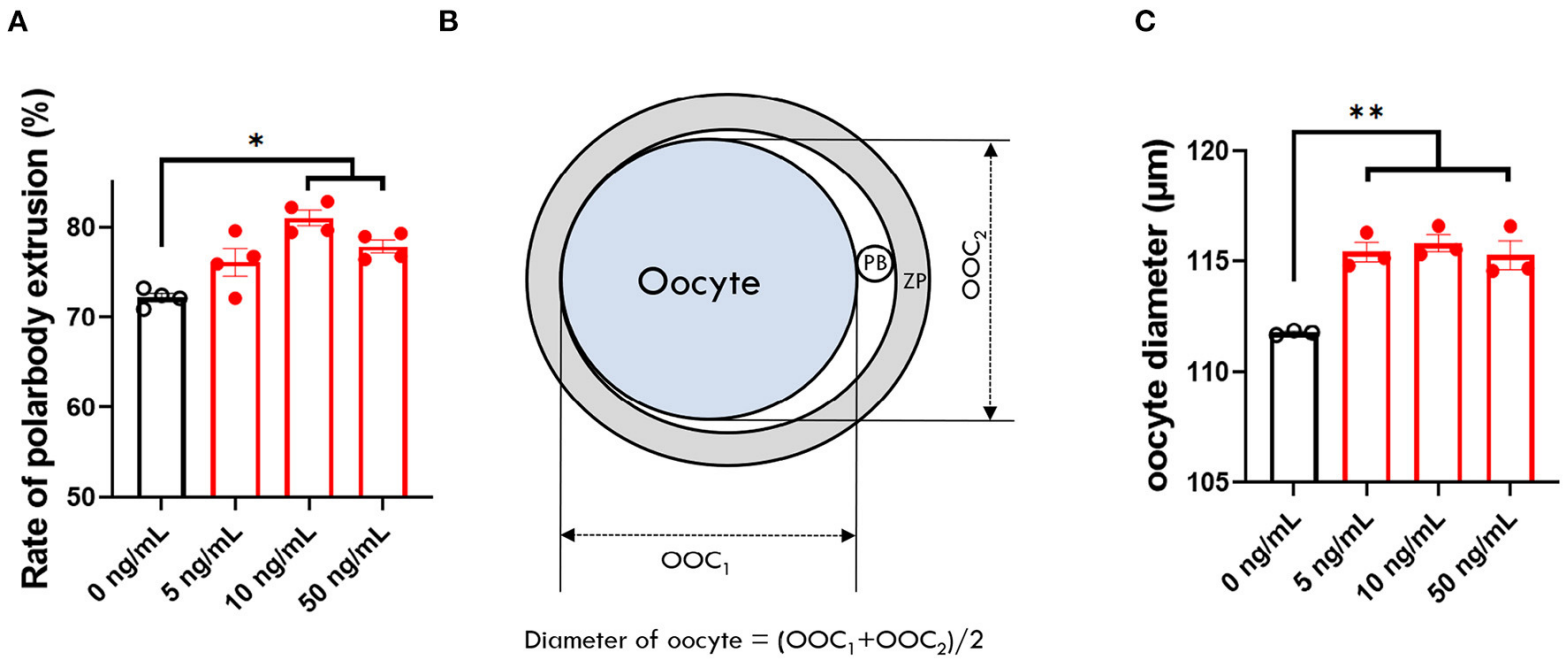
Effect of stem cell factor (SCF) supplementation during *in vitro* maturation (IVM) on nuclear maturation and size of porcine oocytes. **(A)** Quantified rate of polar body extrusion after 42 h of IVM supplemented with various concentrations of SCF. **(B)** A diagram of the measurement of porcine oocyte diameter after IVM modified from Figure 3 of Lee et al. (24). ZP, zona pellucida. PB, polar body. **(C)** Quantified diameter of mature porcine oocytes after 42 h of IVM supplemented with various concentrations of SCF. For all graphs, the value represents the mean ± SEM. Asterisks indicate statistical significance (*p < 0.05 and **p < 0.01). Statistical significance was determined by one-way ANOVA. The experiments were repeated three or four times.

**Figure 3:**
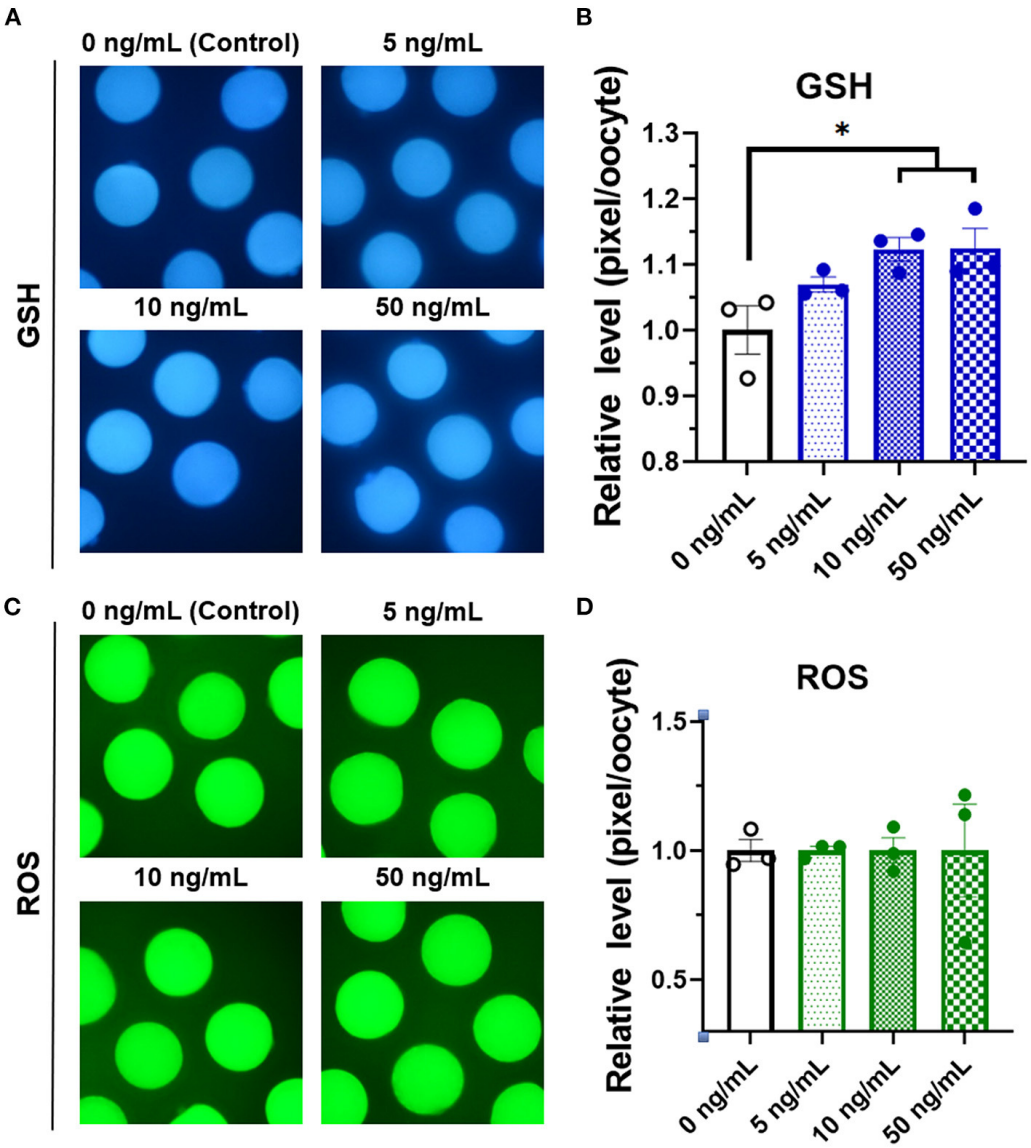
Epifluorescent photomicrographic images of *in vitro* mature porcine oocytes. **(A)** Oocytes were stained with CellTracker Blue to detect intracellular levels of glutathione (GSH). Metaphase II oocytes derived from maturation medium supplemented with SCF (0, 5, 10, and 50 ng/mL). **(B)** The relative levels of intracellular GSH in *in vitro* mature porcine oocytes among the SCF-treated groups. **(C)** Oocytes were stained with 2′,7′-dichlorodihydrofluorescein diacetate to detect intracellular levels of reactive oxygen species (ROS). **(D)** The relative levels of intracellular ROS in *in vitro* mature porcine oocytes among the SCF-treated groups. Asterisks indicate statistical significance (*p < 0.05). Statistical significance was determined by one-way ANOVA. The experiments were performed in triplicate. Total number of examined oocytes: GSH samples, *n* = 240; ROS samples, *n* = 197.

To identify the effect of adding different concentrations of SCF on oocyte maturation during IVM, we evaluated the percentage of oocytes showing the first polar body and diameter of MII oocytes at the end of maturation. The 10 and 50 ng/mL SCF-treated groups showed significantly (*p* < 0.05) higher rates of polar body extrusion (81.00 and 77.83%, respectively) than the control group (72.16%; Figure 2A). Diameters were bigger (*P* < 0.01) in all SCF-supplemented oocytes (115.4, 115.8, and 115.3 μm, respectively) than nontreated oocytes (111.7 μm; Figures 2B,C). To assess cytoplasmic maturation, we examined intracellular GSH and ROS levels in MII oocytes derived from maturation medium supplemented with various concentrations of SCF after IVM. The 10 and 50 ng/mL SCF-treated groups showed significantly increased (*P* < 0.05) intracellular GSH levels compared with the control (Figures 3A,B). However, no significant differences (*p* > 0.05) in ROS generation levels between groups were noted (Figures 3C,D). Thus, we determined that the optimal concentration of SCF supplementation during porcine IVM was 10 ng/mL, which was used in subsequent experiments.

### Effect of SCF/c-Kit Signaling During Porcine IVM on Cumulus Cell Expansion, Mature Oocytes, and Subsequent Embryonic Development After IVF

**Figure 4:**
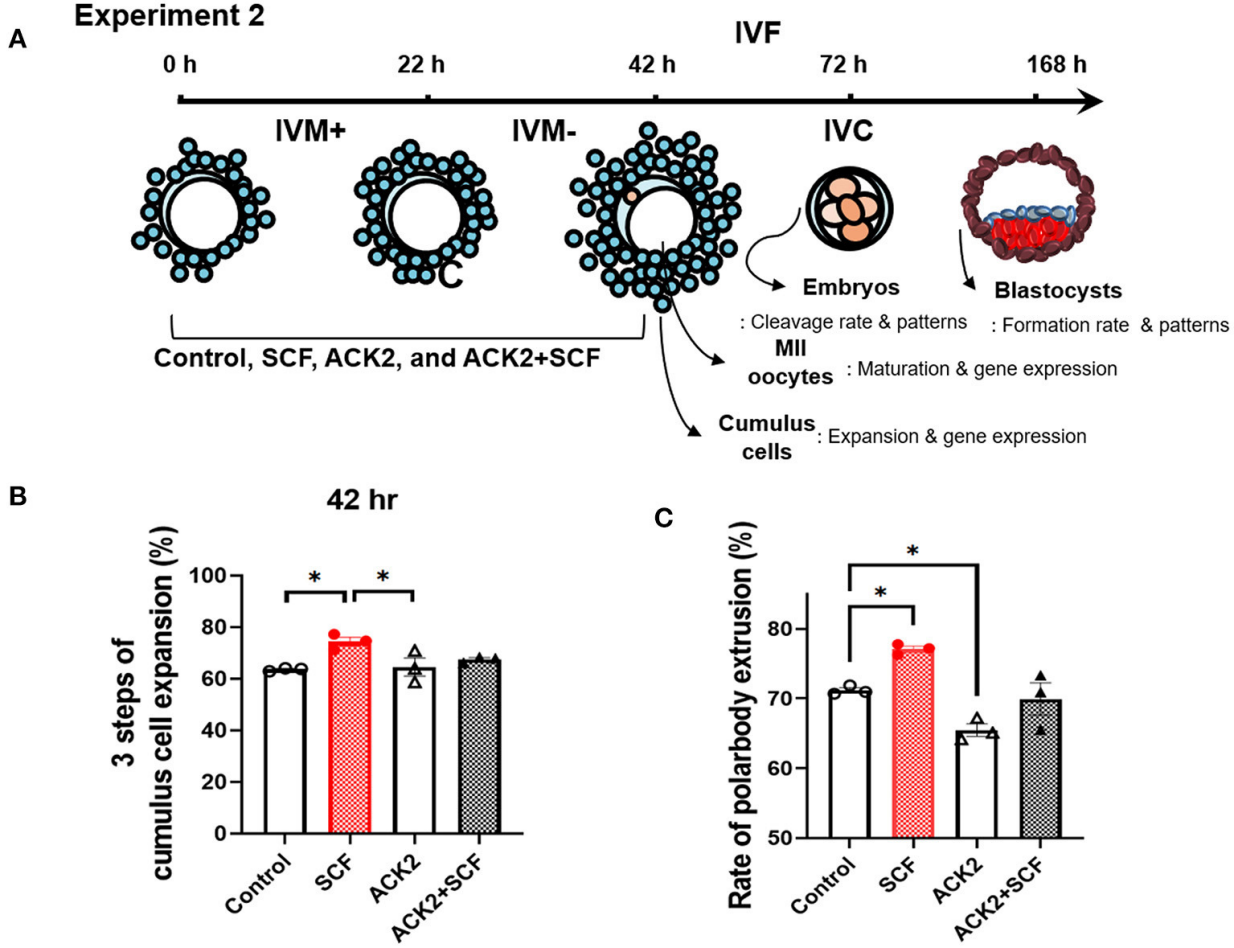
Effect of stem cell factor (SCF)/c-kit signaling during porcine *in vitro* maturation (IVM) on cumulus expansion and nuclear maturation of porcine cumulus-oocyte complexes (COCs). **(A)** Experimental design to examine the effect of SCF/c-kit signaling for porcine oocytes and subsequent developmental competence after *in vitro* fertilization (IVF). IVM+ and IVM– indicate that incubation was in media with or without the eCG hormone, respectively. Inhibition of KIT by ACK2 in porcine oocytes. **(B)** Quantified three steps of cumulus cell expansion after 42 h of IVM supplemented with SCF and/or ACK2. **(C)** Quantified rate of polar body extrusion after 42 h of IVM supplemented with SCF and/or ACK2. For all graphs, the value represents the mean ± SEM. Asterisks indicate statistical significance (*p < 0.05). Statistical significance was determined by one-way ANOVA. The experiments were performed in triplicate.

For experiment 2, as shown in Figure 4A, we evaluated the effects of inhibition of SCF/c-Kit signaling using ACK2 during IVM on porcine COCs and subsequent development after IVF. Porcine oocytes matured in IVM medium were supplemented with SCF, ACK2, and SCF+ACK2 for 42 h. ACK2-treated groups displayed significantly decreased (*p* < 0.05) cumulus cell expansion at 42 h compared with mature COCs of the SCF-treated group (Figure 4B). The polar body extrusion rate of the SCF-supplemented group was significantly (*p* < 0.05) higher (77.08%) than that of the control group (71.18%; Figure 4C). However, the polar body extrusion rate of ACK2-treated groups was significantly (*p* < 0.05) lower (65.48 %) than that of the control group (71.18%; Figure 4C).

**Figure 5:**
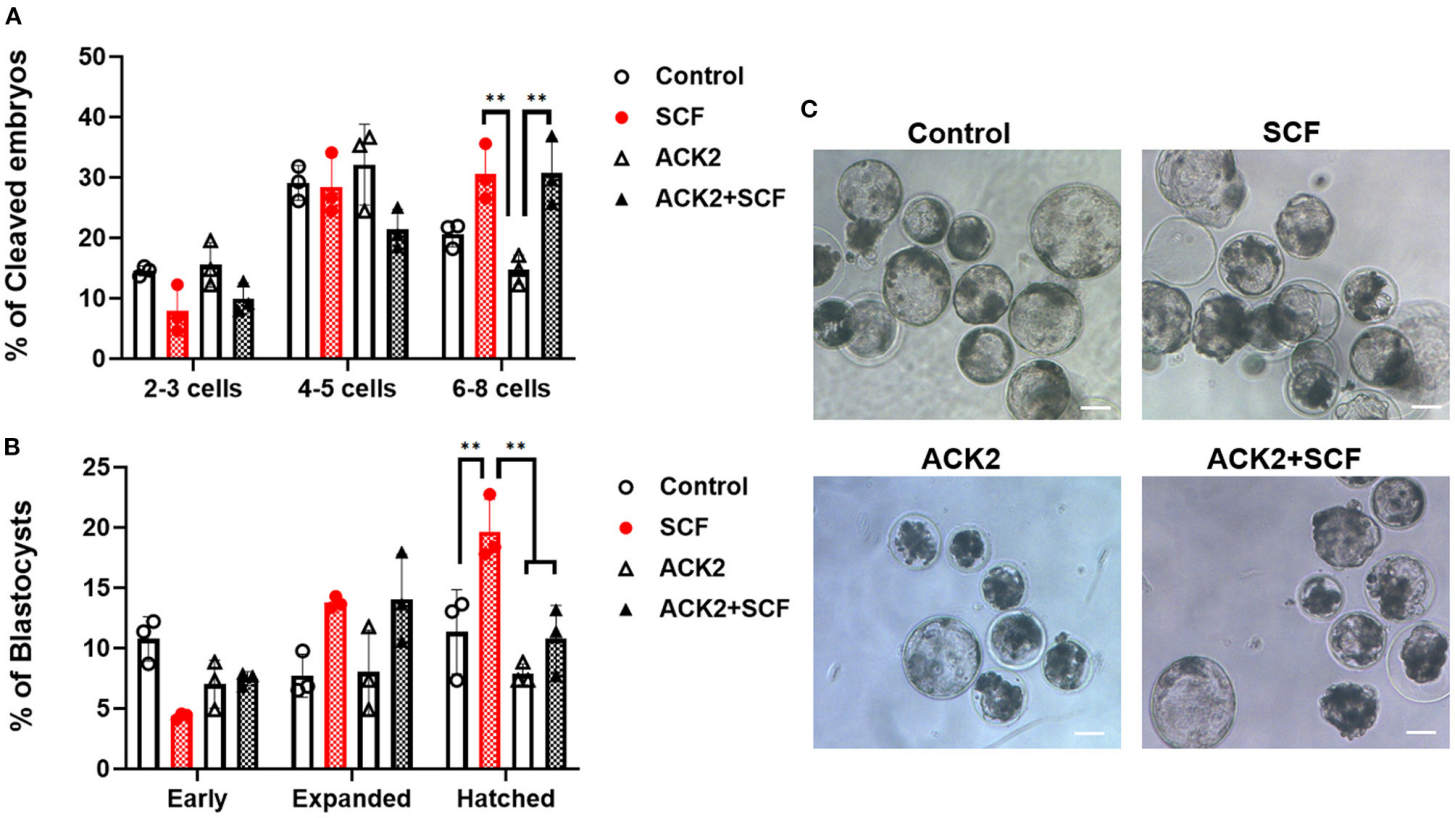
Effect of stem cell factor (SCF)/c-kit signaling during porcine *in vitro* maturation (IVM) on the cleavage pattern of *in vitro* fertilized (IVF) embryos at day 2 **(A)** and the percentage of IVF embryos that developed to the blastocyst stage at day 7 **(B)**. For all graphs, the value represents the mean ± SEM. Asterisks indicate statistical significance (**p < 0.01). Statistical significance was determined by one-way ANOVA. The experiments were performed in triplicate. Early, early blastocyst; Expanded, expanded blastocyst; Hatched, hatched blastocyst. **(C)** Representative morphologies of porcine blastocysts from each group 7 days after IVF. Scale bar = 100 μm.

In the IVF experiment, no significant difference (*p* > 0.05) in cleavage rates was observed between the control, SCF, ACK2, and SCF+ACK2 treated groups (Table 2). The cleavage pattern of 6–8 cell IVF embryos was significantly (*p* < 0.01) decreased in the ACK2-treated group compared with the SCF and SCF+ACK2 treated groups (Figure 5A). However, no significant differences (*p* < 0.05) were observed in the cleavage patterns of 2–3 cell and 4–5 cell IVF embryos. The blastocyst formation rate of IVF embryos was significantly (*p* < 0.05) lower in the ACK2-treated group (24.60 %) than in the SCF-treated group (36.22%; Table 2). When comparing blastocyst formation patterns on day 7, significantly more number of hatched IVF blastocyst (*p* < 0.05) was counted in the SCF-treated group than in the other groups (Figures 5B,C). However, no significant differences (*p* > 0.05) were observed in blastocyst formation patterns of early and expanded blastocysts between the groups.

**Table 2:** Effect of stem cell factor (SCF)/c-kit signaling during porcine *in vitro* maturation (IVM) on embryonic development after *in vitro* fertilization (IVF).

	**Embryo cultured, *n***	**Embryos developed to (** ***n*** **, %)**			
		**≥2-cells**		**Blastocyst**	
Control	131	84	(64.18 ± 0.9)	39	(29.78 ± 1.1)^a, b^
SCF	138	92	(66.78 ± 1.8)	50	(36.22 ± 0.3)^a^
ACK2	116	72	(62.22 ± 1.2)	27	(24.60 ± 3.9)^b^
ACK2 + SCF	121	75	(61.92 ± 1.5)	39	(33.12 ± 0.7)^a, b^

*^a,b^Values with different superscript letters within a column indicate significant differences (p < 0.05). The experiment was repeated three times. Data represent the mean of each replicate ± standard error of the mean. Statistical significance was determined by ANOVA*.

### Contribution of SCF/c-Kit Signaling During Porcine IVM on the Expression of c-Kit Signaling- and Oocyte Secreted Factor-Related Genes in Oocytes

**Figure 6:**
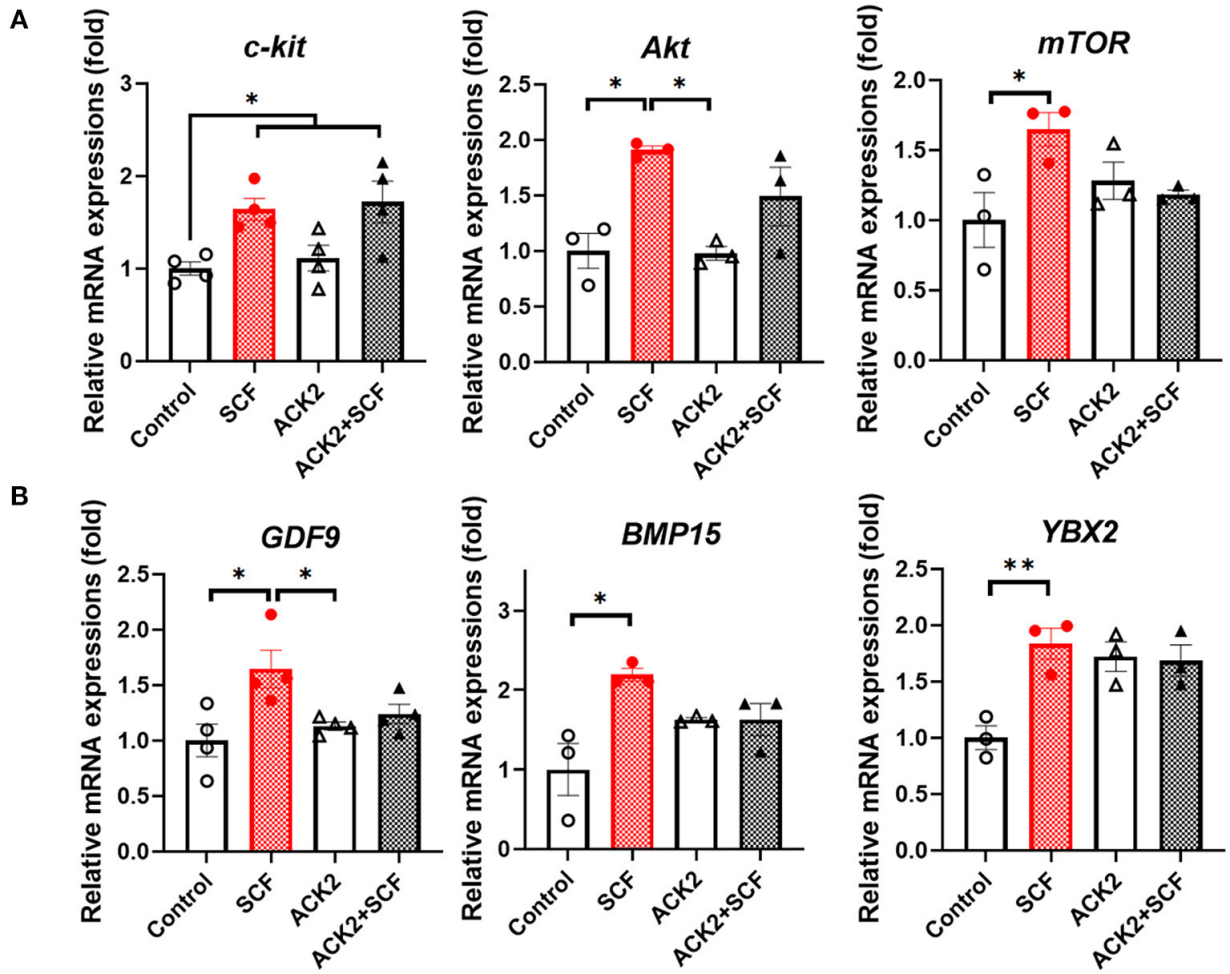
Relative mRNA expression levels in SCF-treated porcine oocytes. **(A)** The mRNA levels of SCF/c-Kit signaling-related genes (*c-kit, Akt*, and *mTOR*). **(B)** The mRNA levels of oocyte secreted factors (*GDF9, BMP15*, and *YBX2*). For all graphs, the value represents the mean ± SEM. Asterisks indicate statistical significance (*p < 0.05 and **p < 0.01). Statistical significance was determined by one-way ANOVA. The experiments were repeated three or four times.

To examine the effect of SCF/c-Kit signaling on the mRNA expression of porcine oocytes, we evaluated c-Kit signaling- and OSF-related genes, including *c-kit, Akt, mTOR, GDF9, BMP15*, and *YBX2*, in each group. As shown in Figure 6A, all c-kit signaling-related transcripts (*c-kit, Akt*, and *mTOR*) were significantly more expressed in oocytes treated with SCF than in the control group (*p* < 0.05). Moreover, *Akt* and *mTOR* levels showed a significant (*p* < 0.05) increase in SCF-treated oocytes compared with the control. Furthermore, all OSF-related transcripts (*GDF9, BMP15*, and *YBX2*) were also significantly (*p* < 0.05) more expressed in oocytes treated with SCF (Figure 6B). Notably, ACK2-treated oocytes during porcine IVM expressed significantly (*p* < 0.05) lower *Akt* and *GDF9* levels than SCF-treated oocytes. Although ACK2-treated oocytes during porcine IVM showed a tendency of lower expression of *c-kit, mTOR*, and *BMP15*, these changes were not significant (*p* > 0.05) compared with the SCF-treated group. In turn, ACK2+SCF-treated oocytes showed a significant increase only in the mRNA levels of *c-kit* compared with the control group.

### Contribution of SCF/c-Kit Signaling During Porcine IVM on the Expression of Apoptosis- and OSF-Related Genes in Cumulus Cells

**Figure 7:**
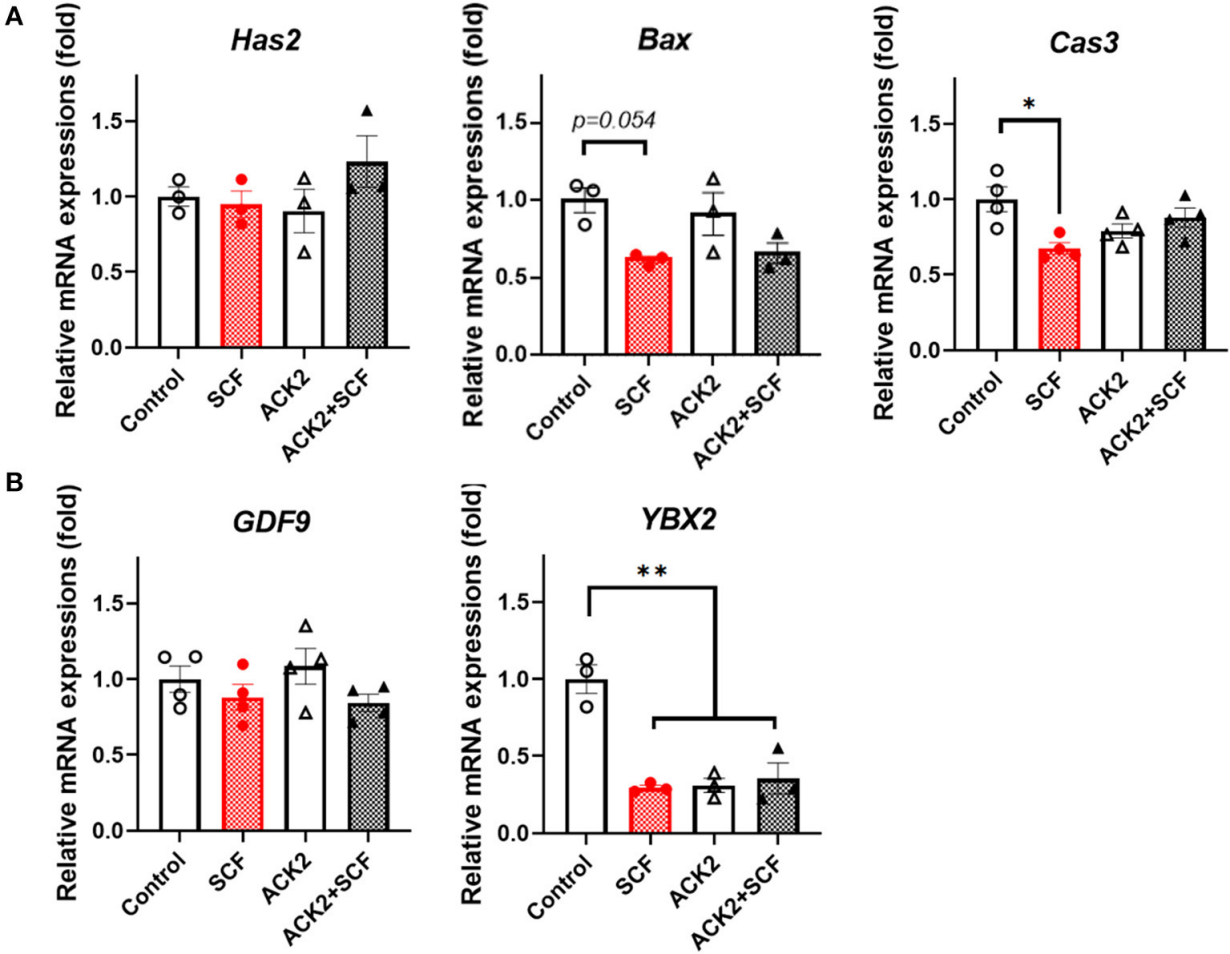
Relative mRNA expression levels in SCF-treated porcine cumulus cells. **(A)** The mRNA levels of cumulus expansion-related *Has2* and apoptosis-related genes (*Bax* and *Cas3*). **(B)** The mRNA levels of oocyte secreted factors (*GDF9*and *YBX2*). For all graphs, the value represents the mean ± SEM. Asterisks indicate statistical significance (*p < 0.05 and **p < 0.01). Statistical significance was determined by one-way ANOVA. The experiments were replicated three or four times.

To examine the effect of SCF/c-Kit signaling on the mRNA expression of porcine cumulus cells, we evaluated apoptosis- and OSF-related genes, including *Has2, Bax, Cas3, GDF9*, and *YBX2*, in each group. As shown in Figure 7A, although SCF-treated cumulus cells showed a tendency to have reduced expression of *Bax* and significantly (*p* < 0.05) lower levels of *Cas3*, no significant difference (*p* > 0.05) in the expression of *Bax* and *Cas3* in ACK2-treated cumulus cells compared with the control was observed. In addition, there were no significant differences (*p* > 0.05) in the expression of *GDF9* in cumulus cells between the groups. Notably, the levels of *YBX2* in ACK2-treated cumulus cells significantly (*p* < 0.01) decreased in all treatment groups compared with the control (Figure 7B).

## Discussion

In this study, we demonstrated that SCF enhances nuclear and cytoplasmic maturation in porcine oocytes *in vitro*. In addition, we showed that SCF downregulates the mRNA levels of proapoptotic-related genes in cumulus cells and upregulates the mRNA levels of OSFs in oocytes, suggesting that this is a mechanism by which SCF/c-Kit signaling controls the maturation of porcine oocytes *in vitro*.

SCF/c-Kit signaling is essential for oocyte growth during early follicular development. It is suggested that SCF produced by granulosa cells binds to the oocyte surface SCF receptor c-Kit, thereby enhancing oocyte growth and follicular development in the ovary. Although SCF/c-Kit signaling promotes ovarian follicle maturation in mice, it is dispensable for primordial follicle activation (29). Although SCF has not been implicated in primordial follicle activation in pigs, it promotes follicle viability *in vitro* (30). SCF is known to promote oocyte growth during the initial stage of growth. The presence of higher amounts of SCF in larger follicles than in smaller follicles (31) suggests that SCF plays a role during the last stages of oocyte growth and maturation. However, there are controversies surrounding the effect of SCF on nuclear maturation after IVM between species and, to our knowledge, there are no published data on the effects of SCF treatment in porcine oocytes matured *in vitro*. Addition of SCF was reported to have no impact on the nuclear maturation of bovine oocytes *in vitro* (16), whereas the extrusion of the first polar body was enhanced in the presence of 50 and 100 ng/mL SCF in buffalo oocytes (17) and 5 and 10 ng/mL SCF in mouse oocytes (15). In mice, although there was no effect of soluble SCF on germinal vesicle breakdown (GVBD), the extrusion of the first polar body was facilitated (15). Another report showed that spontaneous GVBD was observed only in membrane SCF-coincubated mouse oocytes, indicating the acquisition of meiotic competence (32). In the current study, 10 ng/mL soluble SCF improved the nuclear and cytoplasmic maturation of porcine oocytes *in vitro*. These discrepancies may arise from differences between species and can be explained by the variations in experimental methodologies between laboratories. To achieve developmental competence, sufficient cytoplasmic maturation of oocytes is also required, involving the accumulation of mRNA, proteins, substrates, and nutrients (33). Similar to bovine and buffalo oocytes (16, 17), SCF-treated porcine oocytes showed improved cytoplasmic maturation with enhanced levels of GSH, which is an important cytoplasmic factor. Indeed, cytoplasmic GSH levels are essential for sperm chromatin decondensation and male pronucleus formation following sperm penetration (34). Although there were no significant differences in the cleavage rate between SCF– and SCF+ACK2-treated groups following IVF, the percentages of oocytes that reached the 6–8 cell stage and hatched blastocyst stage were significantly (*p* < 0.05) higher in the SCF-treated group. In murine oocytes, the SCF/c-Kit signaling promoted cytoplasmic maturation, measured by the cleavage rate of 2–4 cells following IVF (12). In the current study, although the SCF+ACK2 group showed an ameliorated outcome in terms of the ACK2-induced effect on some parameters, including cleavage patterns of 6–8 cells, no ameliorative effect was observed in most other parameters. This could be explained by the lower binding affinity of soluble SCF for the porcine c-Kit than ACK2. Although further investigations are necessary to analyze this effect, our study showed that blocking c-Kit with ACK2 alone markedly reduced most of the parameters, thereby suggesting that the SCF/c-Kit signals are essential for the porcine IVM system. Overall, these results indicate that SCF/c-Kit signaling during IVM enhances the developmental competence of porcine oocytes.

Oocyte diameter is one of the several parameters used for assessing oocyte quality and meiotic competence (35). During growth and maturation, porcine oocytes increase in diameter from ~30 (premature oocytes in preantral follicles) to 120 μm (fully grown oocytes in preovulatory follicles) (36). Compared with pre-pubertal gilts, cycling female pigs have larger size oocytes (124.7 vs. 113.1 μm) (37). In accordance with previous reports in other species (17, 38), SCF-treated porcine oocytes showed an increased diameter in the present study, which may affect cytoplasmic and nuclear maturation of the oocytes. Oocyte quality can also be assessed by detecting apoptosis. In murine oocytes, SCF has been reported to inhibit apoptosis of oocytes (39) and enhance their survival and growth (12, 32). Indeed, SCF pretreatment enhanced the survival of xenografted porcine oocytes (40). Herein, SCF supplementation during porcine IVM reduced the expression of the proapoptotic factors *Bax* and *Cas3* and prevented the oocyte degeneration triggered by an anti-c-Kit antibody, suggesting that SCF promotes the *in vitro* growth of porcine oocytes by protecting them from apoptosis. However, further studies are necessary to analyze the protein levels of these apoptotic-related factors and the SCF/c-Kit pathway in porcine IVM.

SCF/c-Kit signaling is known to activate the phosphatidylinositol-3-kinase (PI3K)/AKT/mTOR pathway, which is involved in several cellular processes, including survival, proliferation, differentiation, and metabolic homeostasis (41, 42). The increased expression of *c-kit, Akt*, and *mTOR* in SCF-treated oocytes indicates that this pathway plays a role in porcine oocyte maturation. Recent studies have reported functional paracrine communication between the oocyte and surrounding granulosa cells, which is critical for several stages of follicular development (43, 44). During this process, SCF secreted from granulosa cells plays an important role in regulating ovarian cell function, whereas OSFs regulate the activity of granulosa cells (18). Among the OSFs, GDF9 plays an important role in the regulation of oocyte maturation and development and granulosa cell proliferation and differentiation. In rodents, GDF9 inhibits SCF secretion from cumulus cells (45, 46). In this study, SCF treatment increased the levels of *GDF9*, and blockade of the SCF/c-Kit system using ACK2 decreased *GDF9* expression compared with that in the SCF-treated group. Although this finding is not consistent with a report in bovine oocytes (47), which showed no significant difference in GDF9 levels, this expression pattern suggests that SCF/c-Kit signaling during porcine oocyte maturation is involved in paracrine interactions with the secretion of GDF9 from oocytes in pigs. BMP15 is another important OSF, which accumulates in the cortical region of porcine oocytes, and simultaneous expression of GDF9 and BMP15 is critical for meiotic maturation of porcine oocytes (48). In this study, SCF treatment enhanced the expression of BMP15 in porcine oocytes, which is known to prevent cumulus cell apoptosis (49). This result is in accordance with our mRNA expression data of the proapoptotic genes *Bax* and *Cas3* in cumulus cells. However, no mRNA expression of BMP15 was observed in porcine cumulus cells. Among the several Y-box proteins, YBX2 (also called MSY2) is primarily expressed in germ cells (50) and is a highly abundant protein, representing 2% of the total oocyte protein in fully grown oocytes (51). During oogenesis, oocyte growth and meiosis require the timely recruitment of maternal mRNAs, in which YBX2 plays a central role by regulating the stability and translation of maternal mRNAs and protein synthesis (52), with YBX2^−/−^ mice being infertile (53). In agreement with the observations in cattle (47), the levels of *YBX2* were significantly (*p* < 0.01) upregulated in SCF-treated porcine oocytes, suggesting that SCF enhances YBX2 action, contributing to RNA stability and protein synthesis in porcine oocytes.

## Conclusion

In conclusion, SCF supplementation during porcine oocyte IVM has a positive effect on the nuclear and cytoplasmic maturation of oocytes and the expansion of cumulus cells with improved developmental competence to the blastocyst stage following IVF *via* SCF/c-Kit signaling. These effects are associated with the enhanced expression of OSF and reduced apoptosis of cumulus cells. Our findings provide further insights into SCF/c-Kit signaling in porcine COCs during oocyte maturation and subsequent development following IVF.

## Data Availability Statement

The original contributions presented in the study are included in the article/supplementary material, further inquiries can be directed to the corresponding author.

## Author Contributions

EK designed the study, performed the experiments and image analysis, analyzed the data, prepared the figures, was responsible for funding, and wrote and revised the manuscript. LC performed RNA extraction. S-HH designed the study, collected funding, analyzed and interpreted the data, and wrote the manuscript. All authors read and approved the final manuscript.

## Funding

This work was supported by grants from the National Research Foundation of Korea (NRF) funded by the Korean Government (Nos. 2021R1C1C2007132 and 2020R1A2C2008276), the Korea Institute of Planning and Evaluation for Technology in Food, Agriculture, Forestry, and Fisheries (318016-5, 819029-2, 320005-4), and the Global Research and Development Center Program through the NRF, funded by the Ministry of Education, Science and Technology (2017K1A4A3014959).

## Conflict of Interest

The authors declare that the research was conducted in the absence of any commercial or financial relationships that could be construed as a potential conflict of interest.

## Publisher's Note

All claims expressed in this article are solely those of the authors and do not necessarily represent those of their affiliated organizations, or those of the publisher, the editors and the reviewers. Any product that may be evaluated in this article, or claim that may be made by its manufacturer, is not guaranteed or endorsed by the publisher.
